# Characterizing the Emergence of Myeloid‐Derived Suppressor Cell Subsets in a Murine Model of Pulmonary Fibrosis

**DOI:** 10.1096/fj.202500312RR

**Published:** 2025-05-13

**Authors:** Nora Vedder, Philipp Gercke, Nikoleta Lautenschlager, Tobias Brunn, Tim Lange, Jakob Schieb, Charlotte Vetter, Chiel van Geffen, Saeed Kolahian

**Affiliations:** ^1^ German Center for Lung Research (DZL) Universities of Giessen and Marburg Lung Center (UGMLC) Philipps University Marburg Marburg Germany; ^2^ Preclinical Imaging Core Facility, Center for Tumor Biology and Immunology (ZTI) Philipps University Marburg Marburg Germany

**Keywords:** animal model, fibrosis, inflammation, myeloid‐derived suppressor cells, pulmonary fibrosis

## Abstract

The immune system plays a major role in pulmonary fibrosis (PF), a devastating lung disease with limited treatment options. Myeloid‐derived suppressor cells (MDSCs) are immune cells with remarkable immunosuppressive functions. We hypothesized that their anti‐inflammatory activity may dampen PF by inhibiting inflammation and its transition to fibrosis. Here, we studied the emergence of both polymorphonuclear (PMN)‐ and monocytic (M)‐MDSCs in a murine model of PF. We assessed immunological, histopathological, and clinical changes at days 3, 7, 14, and 21 following bleomycin challenge. A comprehensive overview of the role of MDSCs during the acute lung injury and chronic phase of pulmonary fibrosis is provided, along with the effects of MDSCs adoptive transfer and depletion. Inflammation and fibrosis increased over a period of 21 days after bleomycin administration. In the lung, the number of PMN‐MDSCs increased, while M‐MDSCs decreased over the time following bleomycin challenge. Especially, M‐MDSCs showed enhanced suppressive activity on day 3 following bleomycin challenge. Adoptive transfer of PMN‐MDSCs attenuated inflammation and fibrosis development. However, depletion of PMN‐MDSCs did not lead to an exacerbation of PF. Our results suggest that adoptive transfer of PMN‐MDSCs can ameliorate the inflammatory responses and thus the development of fibrosis in a bleomycin‐induced pulmonary fibrosis model.

## Graphical abstract

We hypothesize that myeloid‐derived suppressor cells (MDSCs) might be able to ameliorate pulmonary fibrosis (PF) by inhibiting local inflammation and thus its transition to fibrosis. Various parameters were assessed on days 3, 7, 14, and 21, following bleomycin challenge in mice. Lung function deteriorated, while inflammation and fibrosis increased. Polymorphonuclear (PMN)‐ and monocytic (M)‐MDSCs exhibited different patterns. Adoptive transfer of PMN‐MDSCs attenuated inflammation and fibrosis development. However, depletion of PMN‐MDSCs did not lead to an exacerbation of PF. The graphical abstract and some icons were created with BioRender.com.

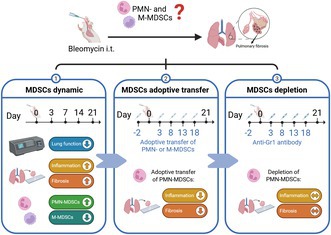

## 1 Introduction

Idiopathic pulmonary fibrosis (IPF) is a chronic interstitial lung disease in which progressive scarring of lung tissue leads to irreversible loss of lung function [1]. Several risk factors for IPF have been identified, including smoking, infections, toxins, radiation, environmental pollutants, aging, genetic factors, gastroesophageal reflux, and autoimmune diseases [1, 2, 3]. However, the process of IPF development remains incompletely elucidated. IPF typically manifests more frequently in men around the age of 60 [4]. Incidence rates in Europe and North America vary from 2.8 to 18 cases per 100 000 individuals per year [5]. The estimated prevalence in the United States ranges from 14.0 to 42.7 per 100 000 individuals [6]. Although data vary from country to country, the incidence appears to be increasing in most countries around the world [7]. Survival rates are low, with a median survival of 2–4 years after diagnosis [5]. Unfortunately, treatment options are limited, with only two FDA‐approved drugs available at the time being pirfenidone and nintedanib. Both drugs lead only to a slower decline in lung function, without halting fibrotic progression [8]. In addition, the use of both drugs is associated with numerous side effects, which can lead to dose reduction or interruption of therapy [9]. To date, lung transplantation remains the only effective therapy for IPF patients [4].

Pathophysiologically, it is assumed that multiple airway cell injuries initiate the disease. This results in a dysregulated wound healing process with excessive deposition of extracellular matrix components (ECMs). Deposition of ECMs is characteristic of IPF and causes a thickening of the alveolar walls, resulting in an impaired lung function [1, 3]. Fibroblasts are recruited to sites of tissue injury and differentiate into myofibroblasts. Myofibroblasts are considered the hallmarks of fibrosis development, being the main producers of ECM. Their expansion is stimulated through various factors and signaling pathways, e.g., tumor growth factor‐β (TGF‐β) or Wnt/β‐catenin [1, 2, 10, 11]. Furthermore, both innate and adaptive immune cells, e.g., macrophages, neutrophils, dendritic cells, and T cell subsets, regulate myofibroblast generation through various mechanisms [2]. The role of immune cells in IPF is complex, with many cells exhibiting both pro‐ and anti‐fibrotic effects. In addition, many signaling pathways have not been fully elucidated, and results are often contradictory. In particular, the role of myeloid‐derived suppressor cells (MDSCs) in IPF remains to be elucidated [1, 2].

MDSCs are a heterogeneous population of immature myeloid cells with potent immunosuppressive and anti‐inflammatory activity [12]. Emerging evidence suggests that MDSCs are involved in infections, acute and chronic inflammation, and autoimmune diseases [13]. They can be divided into two groups, namely, polymorphonuclear MDSCs (PMN‐MDSCs) and monocytic MDSCs (M‐MDSCs). In humans, PMN‐ and M‐MDSCs are defined as CD11b^+^CD14^−^CD15^+^ or CD11b^+^CD14^−^CD66b^+^ and CD11b^+^CD14^+^HLA‐DR^−/lo^CD15^−^, respectively [14]. In contrast, CD11b^+^Ly6C^lo^Ly6G^+^ for PMN‐MDSCs and CD11b^+^Ly6C^hi^Ly6G^−^ for M‐MDSCs are used for characterization in mice [14]. Initially, MDSCs were identified in the context of cancer patients, where they modulate the tumor microenvironment [14]. In addition, they contribute to the immune escape of malignant tumors through their ability to suppress T cells [15]. MDSCs suppress T cells through various mechanisms. The main factors include Arginase 1, inducible nitric oxide synthase, reactive oxygen species, and peroxynitrite. Specifically, Arginase 1 and iNOS are known to inhibit T cell proliferation by depriving L‐arginine and by producing NO, which suppresses T cell function [13, 14, 16]. However, the role of MDSCs in lung fibrosis is not yet clear. Not much is known about the interaction of MDSCs with myofibroblasts. Cheuk et al. reported that especially M‐MDSC‐derived supernatant inhibited the TGF‐β1‐induced differentiation of mesenchymal stem cells to myofibroblasts through interleukin (IL)‐15 secretion [17]. Fernandez et al. observed an accumulation of MDSCs in the peripheral blood and tissue of IPF patients [18]. The increased number of MDSCs was consistent with reduced lung function and an increased number of T regulatory cells [18]. Bryant et al. also detected an increased number of PMN‐MDSCs in the peripheral blood of IPF patients [19]. However, in a mouse model, they observed that an increased number of PMN‐MDSCs led to a reduction in parenchymal fibrosis and an improvement in bleomycin‐induced pulmonary fibrosis [19]. The role of MDSCs has not yet been fully clarified in pulmonary fibrosis and many inflammatory diseases. Further research is needed to determine their significance and how they can be specifically targeted.

In this study, we aim to investigate the role of MDSCs in a murine model of bleomycin‐induced pulmonary fibrosis. Bleomycin‐induced pulmonary fibrosis mimics many pathophysiological features of IPF. As a result, numerous fibrosis mechanisms that also play a role in IPF can be investigated. This enables a deeper understanding of the central aspects of IPF. We hypothesize that the immunosuppressive and anti‐inflammatory activity of MDSCs might be able to ameliorate pulmonary fibrosis by inhibiting local inflammation and thus its transition to lung fibrosis. To further study this hypothesis, we observed the generation, activation, and recruitment of MDSCs over a time span of 21 days following bleomycin administration. In addition, we performed the adoptive transfer of MDSCs as well as MDSCs depletion to gain further insight into the role of MDSCs in the development of pulmonary fibrosis.

## 2 Materials and Methods

### 2.1 Experimental Design

The objective of the study was to investigate the emergence of MDSCs in a murine model of bleomycin‐induced pulmonary fibrosis. For this purpose, the generation, activation, and recruitment of MDSCs were observed over a period of 21 days after administration of bleomycin. Various parameters were assessed on days 3, 7, 14, and 21. In addition, an adoptive transfer of MDSCs and bone marrow, as well as PMN‐MDSC depletion, were performed.

### 2.2 Mice

9‐ to 12‐week‐old female and male C57BL/6N mice were obtained from Charles River Laboratories (Sulzfeld, Germany). Mice were accommodated in individually ventilated cages with a light/dark cycle of 12 h; food and water were provided *ad libitum*. All animal studies were reviewed and approved by the Regierungspräsidium Giessen and complied with the guidelines of the German law of protection of animal life (TierSchVersV, G8/2022).

### 2.3 Induction of Pulmonary Fibrosis

Mice were randomly assigned into two groups and received either 1.5 U/kg bleomycin (BleoCell, Stada Arzneimittel AG, Bad Vibel, Germany) in 50 μL of 0.9% normal saline (Fresenius Kabi Deutschland GmbH, Bad Homburg, Germany) or 50 μL of 0.9% normal saline intratracheally (i.t.) on day 0. I.t. application was performed under anesthesia with ketamine (50 mg/kg¸ Serumwerk Bernburg Tiergesundheit GmbH, Bernburg Germany) and medetomidine (1 mg/kg, Orion Pharma AG, Espoo, Finnland) injected intraperitoneally (i.p.). After i.t. application, mice were recovered by antagonization using 1.5 mg/kg atipamezole (Orion Pharma AG, Espoo, Finnland) i.p.

### 2.4 In Vivo Respiratory Mechanics

**FIGURE 1:**
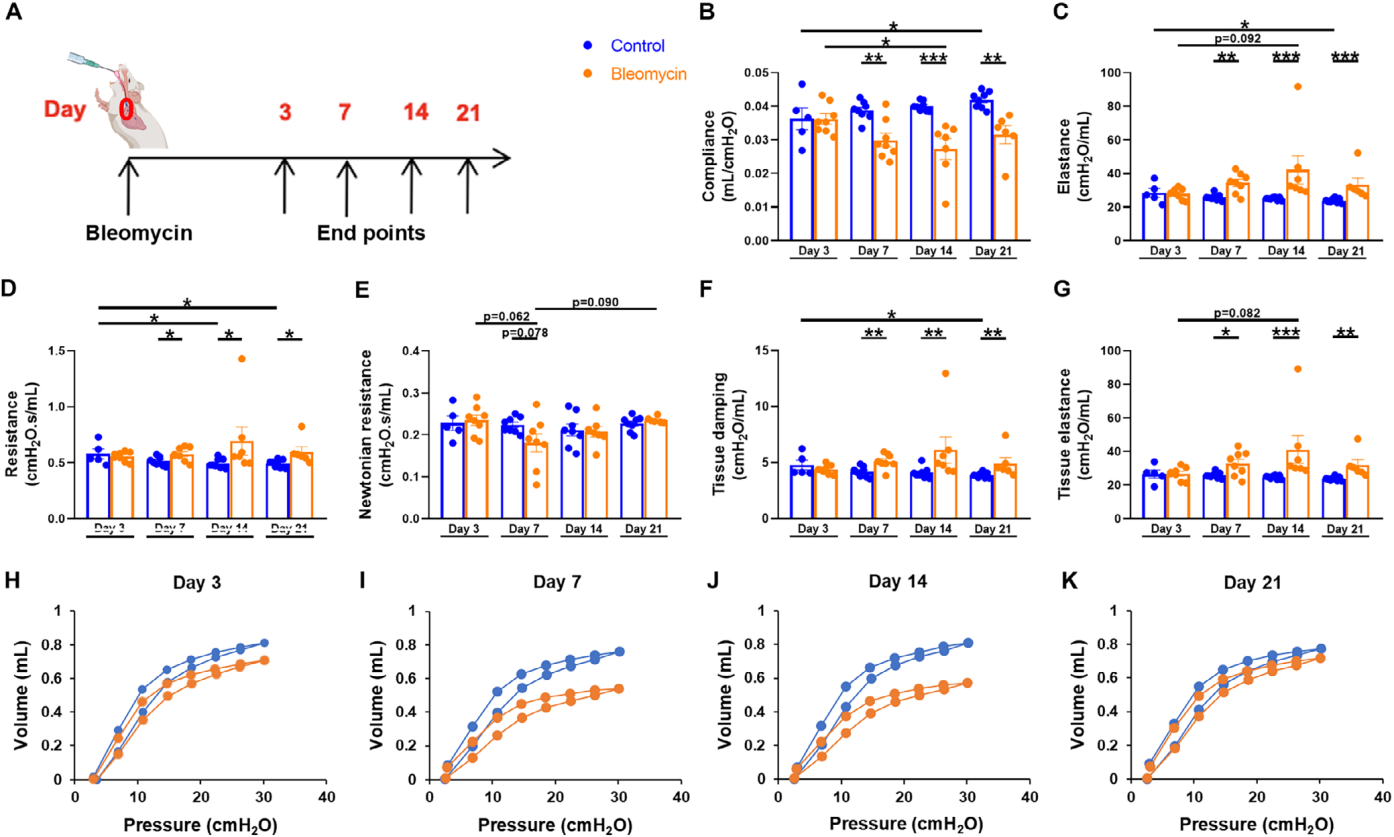
The effect of a murine model of bleomycin‐induced pulmonary fibrosis on in vivo lung function measurements on days 3, 7, 14, and 21. (A) Timeline of the murine model of bleomycin‐induced pulmonary fibrosis. 9‐ to 12‐week‐old C57BL/6N mice received 1.5 U/kg bleomycin or 0.9% normal saline intratracheally (i.t.) on day 0. On days 3, 7, 14, and 21 after bleomycin challenge, lung function and immunological parameters were investigated. Various parameters were obtained during an in vivo respiratory lung function measurement, such as (B) compliance (C_rs_), (C) elastance (E_rs_), (D) resistance (R_rs_), (E) Newtonian resistance (R_n_), (F) tissue damping (G), (G) tissue elastance (H) and PV curves. Representative PV curves for (H) day 3, (I) day 7, (J) day 14, and (K) day 21. Data were obtained from 8 independent experiments, *n* = 5–8. Results are shown as mean ± SEM. Statistical analysis was performed within the control and the bleomycin group between different days with one‐way ANOVA followed by Tukey's multiple comparisons test or with Kruskal–Wallis test followed by Dunn's multiple comparisons test. For the comparison of the control and the bleomycin group of the same day, unpaired two‐tailed Student's t‐tests or Mann–Whitney test was used. If data followed normal distribution, one‐way ANOVA or unpaired two‐tailed Student's t‐tests was performed. Otherwise, Kruskal–Wallis test or Mann–Whitney test was used. Significance was defined as follows: *p < 0.05, **p < 0.005, ***p < 0.001. Nonsignificant results are listed if *p*‐value is > 0.05 and ≤ 0.099.

On days 3, 7, 14, and 21 after bleomycin treatment (Figure 1A), an in vivo lung function measurement was performed using a flexiVent system (SCIREQ, Montreal, Canada). Mice were anesthetized by i.p. injection of 120 mg/kg ketamine and 1 mg/kg medetomidine. Once a surgical level of anesthesia was achieved, tracheotomy was performed with an 18 G cannula, and mice were connected to the flexiVent machine. Mechanical ventilation was maintained with a respiratory rate of 150 breaths/min, a tidal volume of 10 mL/kg body weight, and a positive end expiratory pressure of 3 cm H_2_O. 0.8 mg/kg Pancuronium (Inresa Arzneimittel GmbH, Freiburg, Germany) was injected to prevent spontaneous breathing. Subsequently, mouse mechanics scan was used to perform SnapShot‐150 perturbations, Quick‐Prime 3 s perturbations, and pressure‐volume (PV) curves. Before each measurement, deep inflation was performed to recruit closed airways and standardize lung volume. Various measurements were obtained, such as compliance (C_rs_), which describes the distensibility of the lungs and thus their elastic properties; elastance (E_rs_), which is a measure for elastic stiffness and the reciprocal value of compliance; resistance (R_rs_), which represents the total resistance of the respiratory system; Newtonian resistance (R_n_), which describes airway resistance, especially in the large conducting airways; tissue damping (G), which reflects the ability of a tissue to regain its original shape; and tissue elastance (H), which represents the amount of energy that remains in the alveoli. PV curves were performed to obtain various parameters, such as quasi‐static compliance (C_st_), which describes the intrinsic elastic properties of the lungs and chest wall at rest; quasi‐static elastance (E_st_), which is the reciprocal value of C_st_; shape parameter (K), which is a volume‐independent parameter that can be determined from the slope of the PV curve; area, which describes the area between the inflation and deflation legs of the PV curve; and estimate of the inspiratory capacity (A). Only measurements with a coefficient of determination (COD) > 0.9 were used for analysis.

### 2.5 Bronchoalveolar Lavage Fluid (BALF) Collection

After flexiVent measurements, mice were sacrificed and BALF was obtained. Lungs were flushed three times with 0.8 mL PBS supplemented with 0.2% 0.5 M EDTA (Merck KGaA; Sigma‐Aldrich, Taufkirchen, Germany). BALF was centrifuged, and the supernatant was stored at −20°C. The cell pellet was prepared into a single‐cell suspension from which 1 × 10^5^ cells were extracted for Cytospin analysis (Thermo Shandon Ltd., Cheshire, UK). Cells were stained with modified Giemsa staining (Carl Roth GmbH + Co. KG, Karlsruhe, Merck, Germany), and a total of 400 cells were counted under a light microscope and classified according to neutrophils, macrophages, lymphocytes, and eosinophils.

### 2.6 Single Cell Preparation for Flow Cytometry

After BALF collection, lungs and spleen were harvested. Lungs were minced and incubated for 45 min at 37°C in a mixture of Hank's Balanced Salt Solution [HBSS (Thermo Fisher, Scientific, Waltham, MA, USA)], 25 U/mL DNAse I (Thermo Fisher, Scientific) and 200 U/mL collagenase type 4 (Merck, KGaA; Sigma‐Aldrich). Organs were mashed through 70 μm and 40 μm strainers, and red blood cells were lysed with ACK lysis buffer (155 mM ammonium chloride, 10 mM potassium bicarbonate and 0.1 mM EDTA; all from Merck KGaA; Sigma‐Aldrich). A portion of the single‐cell suspension was fixed for flow cytometry.

### 2.7 Flow Cytometry

Single cell suspensions of lung, spleen, and BALF were stained with APC‐CD11b (M1/70), PE/Cy7‐Ly6C (HK1.4) and FITC‐Ly6G (1A8) to characterize MDSCs. T cells were stained with APC‐CD4 (GK1.5), PE‐IL‐4 (11B11), PerCP‐IL‐17A (TC11‐18H10.1), FITC‐IFN‐γ (XMG1.2), FITC‐CD25 (3C7), PE‐FoxP3 (150D) and PE‐CD69 (H1.2F3) antibodies (all from BioLegend, San Diego, USA) to distinguish different T cell subsets. Stained cells were analyzed by flow cytometry using FACS Canto II (BD Biosciences, Heidelberg, Germany) and data was analyzed with FlowJo software version 10.7.1 (BD Biosciences; FlowJo LLC, Ashland, OR, USA).

### 2.8 MDSC Isolation

After single cell preparation, MDSC isolation was performed with the single cell suspension of the lung using the MACS Isolation Kit (Miltenyi Biotec, Bergisch Gladbach, Germany) following the manufacturer's instructions. Briefly, PMN‐MDSCs were isolated by positive selection based on the Ly6G marker. From the remaining suspension, M‐MDSCs were labeled with the marker Gr1 and selected via positive selection.

### 2.9 MDSC Suppression Assay

After MDSC isolation, PMN‐ and M‐MDSCs were co‐cultured with 1 × 10^5^ 5,6‐Carboxyfluorescein diacetate succinimidyl ester (CFSE)‐labeled CD4^+^ T cells at different ratios (MDSC:T cell at 1:1, 1:2, 1:4, and 1:8). Briefly, CD4^+^ T cells were obtained from the spleens of naive C57BL/6N mice, isolated with a T cell isolation kit (Miltenyi Biotec), and stained with 1 μmol/L CFSE (Biolegend) according to the manufacturer's instructions. CFSE‐stained T cells were stimulated with 100 U/mL IL‐2 (BioLegend), 50 μM 2‐mercaptoethanol (Thermo Fisher Scientific) and CD3ɛ‐ and CD‐28 biotin‐loaded anti‐Biotin MACSiBead particles (Miltenyi Biotec) before plating for co‐culture. Cells were cultured for 86 h at 37°C in RPMI 1640 medium (anprotec, Bruckberg, Germany), supplemented with 10% fetal bovine serum (Capricorn Scientific GmbH, Ebsdorfergrund, Germany), 2 mM of L‐glutamine (anprotec/Capricorn Scientific GmbH) and 1% penicillin–streptomycin (anprotec/Capricorn Scientific GmbH). T cells were stained with an APC‐CD4 antibody (GK1.5, Biolegend), and flow cytometry was performed to assess the proliferation of CFSE‐labeled CD4^+^ T cells. The proliferation was normalized to the positive control of each experiment (stimulated CD4^+^ T cells, without MDSCs, set to 100%).

### 2.10 Lung Histopathology

After harvesting the lungs, the left diaphragmatic lobe was fixed in 4% paraformaldehyde (Carl Roth GmbH + Co. KG). Briefly, the lungs were dehydrated, embedded in paraffin, cut into 4–5 μm sections, and stained with Hematoxylin and Eosin (H&E) and Masson's trichrome (MT) staining (both Carl Roth GmbH + Co. KG) according to manufacturer's protocols. The modified Ashcroft Score [20] was used to assess pulmonary fibrosis, and the Szapiel score [21] was used to assess alveolar inflammation.

### 2.11 Enzyme‐Linked Immunosorbent Assay (ELISA)

ELISA for TGF‐β1 was carried out according to the manufacturer's protocols (DY1679, R&D Systems, Minneapolis, USA) in BALF supernatants.

### 2.12 MDSC Adoptive Transfer

1.5 U/kg bleomycin (BleoCell, Stada Arzneimittel AG) in 50 μL of 0.9% normal saline (Fresenius Kabi Deutschland GmbH) was administered i.t. to 9‐ to 12‐week‐old female and male C57BL/6N mice used for adoptive transfer. Since our initial experiments showed that MDSCs exhibited the strongest suppressive activity 3 days after bleomycin administration, we decided to perform adoptive transfer on the third day after bleomycin challenge (Figure 3H,L). Mice treated with bleomycin 3 days earlier were sacrificed, and bone marrow from the hind legs and lungs was collected. PMN‐ and M‐MDSCs were isolated from the lungs using the MACS Isolation Kit (Miltenyi Biotec). APC‐CD11b, PE/Cy7‐Ly6C, FITC‐Ly6G, and 7‐AAD (all from BioLegend) were used as markers to characterize MDSCs. For characterization of the bone marrow, APC‐CD11b and 7‐AAD (both BioLegend) were used. Purity and viability were assessed by flow cytometry. 2 × 10^6^ PMN‐MDSCs and 2 × 10^5^ M‐MDSCs were dissolved in 200 μL PBS and injected i.p. in C57BL/6N mice receiving adoptive transfer. The number of adoptively transferred PMN‐ and M‐MDSCs differed since PMN‐MDSCs comprise more than 70% of the total MDSC population in peripheral lymphoid organs [22, 23]. In addition, in line with the 3R principles of research on animals, we isolated PMN‐ and M‐MDSCs for adoptive transfer from a single mouse and were able to obtain significantly more PMN‐ than M‐MDSCs from the lung. To reflect the physiological situation as closely as possible, we transferred PMN‐ and M‐MDSCs in different cell numbers. For the adoptive transfer of bone marrow, mice received 2 × 10^5^ or 2 × 10^6^ bone marrow cells in 200 μL PBS i.p. The results of both the 2 × 10^5^ and 2 × 10^6^ bone marrow adoptive transfer are combined in one diagram, as no difference was found between the groups. The control mice received 200 μL PBS. Adoptive transfer was performed five times, 2 days before bleomycin administration and then on days 3, 8, 13, and 18 (Figure 5A). The mice were sacrificed on day 21, and lungs, spleen, and BALF were collected for the above‐mentioned experiments.

### 2.13 PMN‐MDSC Depletion

9‐ to 12‐week‐old female and male C57BL/6N were randomized into two different groups. All mice received 1.5 U/kg bleomycin (BleoCell, Stada Arzneimittel AG) in 50 μL of 0.9% normal saline (Fresenius Kabi Deutschland GmbH) on day 0. One group received 200 μg of an anti‐Gr1 antibody (clone 1A8, BioXCell, Lebanon, USA) to deplete the PMN‐MDSCs. The control group received 200 μg of an isotype antibody (clone LTF‐2, BioXCell). The antibody was injected i.p. five times at an interval of 5 days (Figure 6A). On day 21, mice were sacrificed and lungs, spleen, and BALF were collected for the above‐mentioned experiments.

### 2.14 Statistical Analysis

Statistical analyses were performed using GraphPad Prism version 8.0.1 (San Diego, USA). The Shapiro–Wilk test was used to examine whether the data were normally distributed. Normally distributed data were analyzed by one‐way analysis of variance (ANOVA) followed by Tukey's multiple comparison test or by unpaired two‐tailed Student's t‐tests. Not normally distributed data were analyzed by the Kruskal–Wallis test followed by Dunn's multiple comparison test or by the Mann–Whitney test. Data are presented as mean ± SEM. A *p*‐value of < 0.05 was considered statistically significant.

## 3 Results

### 3.1 Bleomycin Challenge Induced Characteristic Pulmonary Fibrosis Respiratory Malfunction In Vivo

Respiratory mechanics were assessed in vivo on days 3, 7, 14, and 21 after bleomycin administration using flexiVent (Figure 1A). Compared to the control group, the group receiving bleomycin showed a significant deterioration in lung function in almost all measured parameters, which was most pronounced on day 14 (Figure 1B–G). Bleomycin was found to significantly reduce the compliance, especially on days 7, 14, and 21 (Figure 1B). Accordingly, a significant increase in elastance was observed on the same days (Figure 1C). Resistance was significantly increased on days 7, 14, and 21 after bleomycin challenge (Figure 1D). Tissue damping, tissue elastance, quasi‐static compliance, quasi‐static elastance, the shape parameter, and the estimate of inspiratory capacity showed the same pattern of deterioration of parameters characteristic of pulmonary fibrosis in the bleomycin group from day 7 onwards (Figures 1F,G and S1A–C,E). No significant differences were observed for other parameters such as Newtonian resistance and area (Figures 1E and S1D). In addition, the PV curves in the bleomycin group showed a rightward shift typical of fibrosis, which was most pronounced on day 14. On day 21, we observed a leftward shift in the bleomycin group back to the PV curve of the control group (Figure 1H–K).

### 3.2 Inflammation and Fibrosis Were Significantly Increased Over a Period of 21 Days After Administration of Bleomycin

**FIGURE 2:**
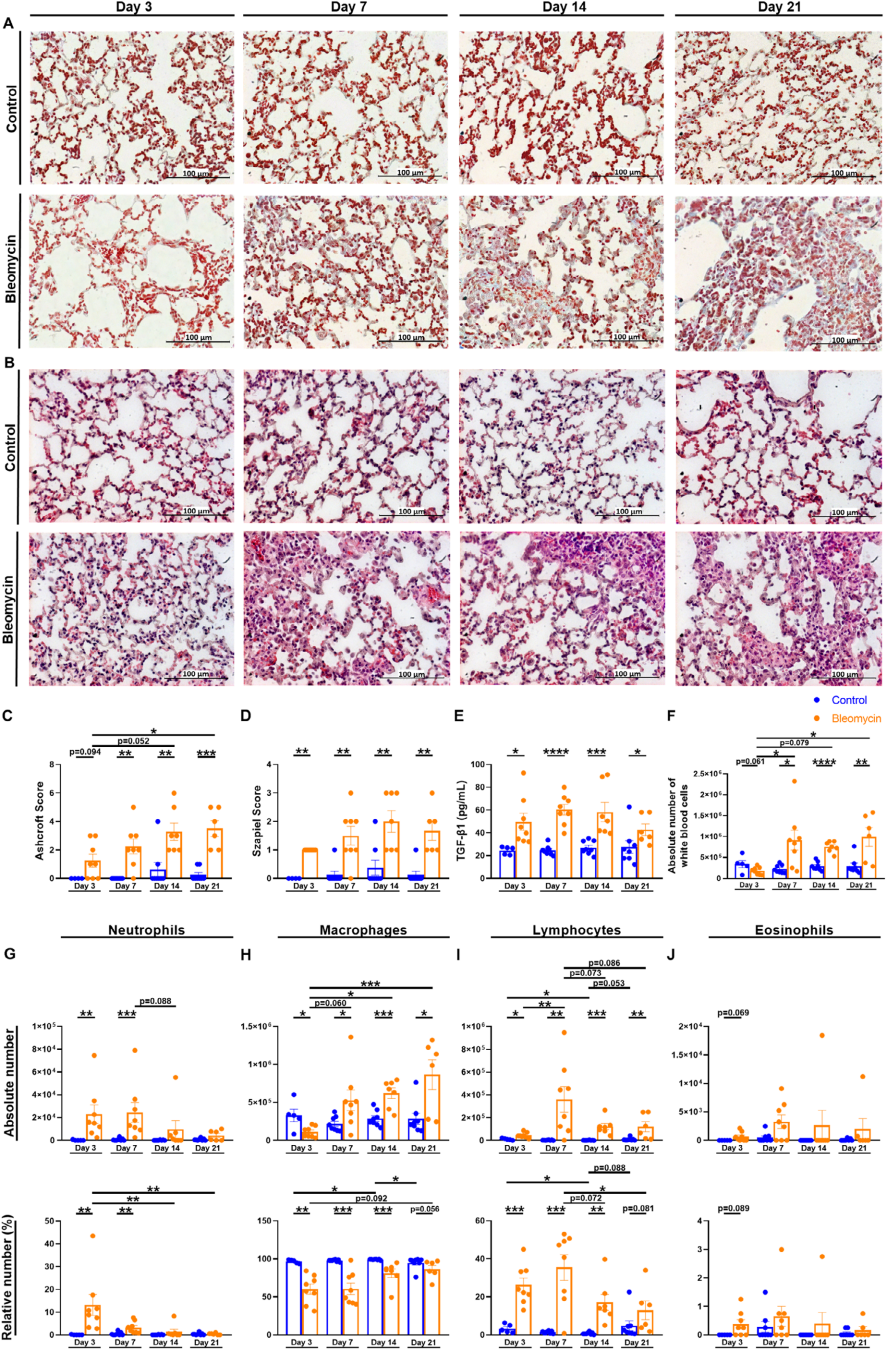
The effect of a murine model of bleomycin‐induced pulmonary fibrosis on inflammation and fibrosis development on days 3, 7, 14 and 21. 9‐ to 12‐week‐old C57BL/6N mice received either 50 μL of 0.9% normal saline or 50 μL of 1.5 U/kg bleomycin i.t. on day 0. (A–D) On days 3, 7, 14 and 21 the left diaphragmatic lobe was collected for histopathological analysis. Representative histological images (20× magnification) of the control and the bleomycin group stained with (A) MT or (B) H&E. (C) Pulmonary fibrosis was assessed with the Ashcroft score. (D) Alveolar inflammation was assessed using the Szapiel score. (E–J) BALF was collected and processed for a differential cell count, and the supernatant was stored. (E) TGF‐β1 concentration was determined with an ELISA from the BALF supernatant. (F) Data shows the absolute number of white blood cells. The absolute cell number and the relative cell number is presented for (G) neutrophils, (H) macrophages, (I) lymphocytes and (J) eosinophils. Data was obtained from 8 independent experiments, *n* = 4–8. Results are shown as mean ± SEM. Statistical analysis was performed with one‐way ANOVA followed by Tukey's multiple comparisons test or unpaired two‐tailed Student's t‐tests if data followed normal distribution. Otherwise Kruskal–Wallis test followed by Dunn's multiple comparisons test or Mann–Whitney test was used. Significance was defined as follows: *p < 0.05, **p < 0.005, ***p < 0.001, ****p < 0.0001. Nonsignificant results are listed if *p*‐value is > 0.05 and ≤ 0.099.

To evaluate histopathological changes associated with pulmonary fibrosis, the diaphragmatic lobe of the left lung was collected on days 3, 7, 14, and 21 after bleomycin administration. MT and H&E staining were carried out (Figure 2A,B). The Modified Ashcroft Score [20] was used to assess pulmonary fibrosis based on MT staining. In the bleomycin group, the scores increased steadily until day 21, and significant differences were evident from day 7 onwards compared to the control group (Figure 2C). These increased values were indicative of the development of pulmonary fibrosis. Strikingly, fibrosis development visibly increased from day 3 to day 21 (Figure 2A). The first changes observed were an enlargement of the alveoli and a thickening of the alveolar septa, as can be seen on day 3 and day 7, followed by a successive destruction of the lung structure and the development of fibrotic masses, which are stained blue and can be observed starting from day 7 (Figure 2A) [20]. In addition, the Szapiel score [21] was used to assess inflammation in the lung based on H&E staining (Figure 2B). Similar to fibrosis development, higher scores were observed in the bleomycin group compared to the control group. Significant differences were detected from day 3 onwards (Figure 2B,D). The scores increased over a period of 21 days, indicating increased inflammation (Figure 2B,D). The inflammation was characterized by an increasing infiltrate of mononuclear cells, ranging from a thickening of the alveolar septum, as seen from day 3, to a diffuse alveolitis from day 7 [21].

To determine the TGF‐β1 concentration, an ELISA was performed with BALF supernatant collected on days 3, 7, 14, and 21. Notably, the TGF‐β1 concentration was already significantly higher on day 3 compared to the control group (Figure 2E). TGF‐β1 levels peaked on day 7 and remained significantly elevated compared to the control group until day 21 (Figure 2E).

To elucidate the alterations of white blood cells after bleomycin administration, BALF was collected for analysis on days 3, 7, 14, and 21. In the bleomycin group, there was a significant increase in the absolute number of white blood cells from day 7, which persisted until day 21 (Figure 2F). This is consistent with the increase in inflammation, fibrosis, and TGF‐β1 in the bleomycin group. Moreover, a differential white blood cell count was performed, which revealed a significant augmentation in neutrophils number within BALF on days 3 and 7, followed by a notable decrease (Figure 2G). Conversely, a continuous increase was observed in macrophages up to day 21 (Figure 2H). This suggests a shift from neutrophilic inflammation to an inflammatory response characterized by an amplified presence of macrophages. Additionally, the lymphocyte count was significantly increased in the bleomycin group on all four measurement days compared to the control group, with the most pronounced elevation seen on day 7 (Figure 2I). No significant differences in eosinophils number were observed (Figure 2J).

### 3.3 In the Lung, PMN‐MDSCs Increased While M‐MDSCs Decreased Over the Time After Administration of Bleomycin

**FIGURE 3:**
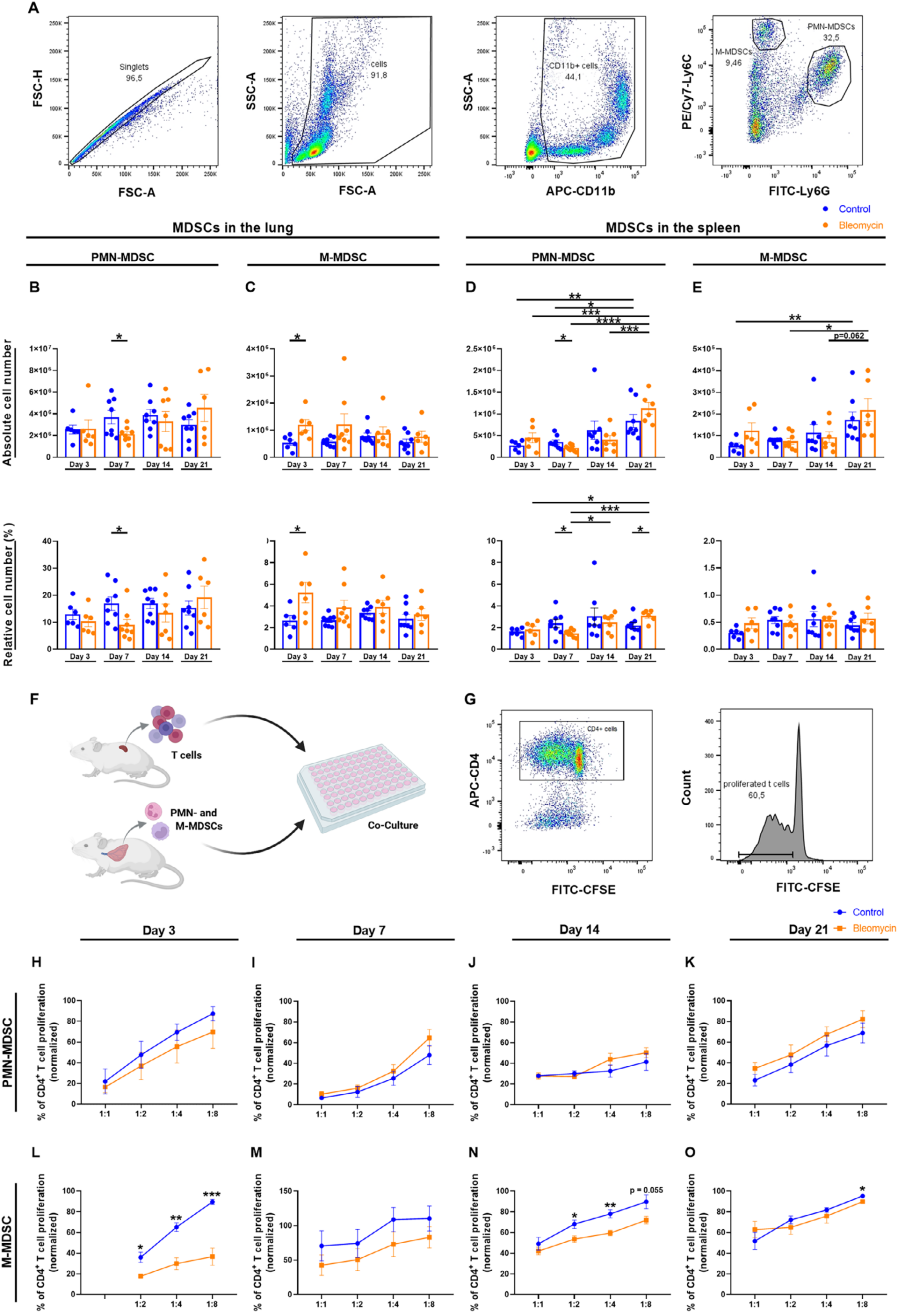
MDSC subtyping in lung and spleen and immunosuppressive activity of MDSCs on CD4^+^ T cell proliferation on days 3, 7, 14, and 21. 9‐ to 12‐week‐old C57BL/6N mice received either 50 μL of 0.9% normal saline or 50 μL of 1.5 U/kg bleomycin i.t. on day 0. (A–E) Flow cytometry was performed on days 3, 7, 14, and 21. (A) Representative gating strategy for PMN‐ and M‐MDSCs. Absolute and relative cell number in the lung shown for (B) PMN‐MDSCs, (C) M‐MDSCs and in the spleen for (D) PMN‐MDSCs, (E) M‐MDSCs. (F–O) (F) CFSE‐labeled CD4^+^ T cells were co‐cultured with PMN‐ or M‐MDSCs at different ratios before flow cytometry was performed. (G) Representative gating strategy to determine CD4^+^ T cell proliferation. Suppressive activity of PMN‐MDSCs on days (H) 3, (I) 7, (J) 14, and (K) 21 and for M‐MDSCs on days (L) 3, (M) 7, (N) 14 and (O) 21. Data represent percentages of proliferating CD4^+^ T cells, normalized to the positive control of each experiment. Data was obtained for (A–E) from 8 independent experiments, including two replicates, *n* = 6–8, and for (F–O) from 1 to 2 independent experiments, *n* = 4–8. Results are presented as mean ± SEM. Statistical analysis was performed with one‐way ANOVA followed by Tukey's multiple comparisons test or unpaired two‐tailed Student's t‐tests if data followed a normal distribution. Otherwise, the Kruskal–Wallis test followed by Dunn's multiple comparisons test or Mann–Whitney test was applied. Significance was defined as follows: *p < 0.05, **p < 0.005, ***p < 0.001, ****p < 0.0001. Nonsignificant results are listed if the *p*‐value is > 0.05 and ≤ 0.099.

To investigate the generation and recruitment of MDSCs, the number of PMN‐ (CD11b^+^Ly6C^lo^Ly6G^+^) and M‐MDSCs (CD11b^+^Ly6C^hi^Ly6G^−^) in lung and spleen was assessed by flow cytometry on days 3, 7, 14, and 21 (Figure 3A). In the lung, we observed a significantly lower number of PMN‐MDSCs on day 7 in the group that received bleomycin than in the control group. However, there appears to have been an increase in PMN‐MDSCs in the control group on days 7 and 14, which may have contributed to this significance. Nevertheless, this increase was not significant compared to day 3. Subsequently, we detected a trend towards rising numbers in the bleomycin group until day 21 (Figure 3B). In contrast, the number of M‐MDSCs in the bleomycin group was significantly higher on day 3. Thereafter, there was a trend towards gradually decreasing cell counts, which were similar to those of the control group on day 21 (Figure 3C). In the spleen, the number of PMN‐MDSCs was significantly reduced in the bleomycin group compared to the control group on day 7. No changes were detected for M‐MDSCs in the first few days. Afterwards, a continuous increase in the absolute cell number of PMN‐ and M‐MDSCs was observed in both the bleomycin and the control group, which persisted until day 21 (Figure 3D,E).

### 3.4 M‐MDSCs in Particular Showed Enhanced Suppressive Activity After Administration of Bleomycin

To analyze the suppressive activity of MDSCs, CFSE‐labeled CD4^+^ T cells were co‐cultured together with PMN‐ and M‐MDSCs at different ratios (Figure 3F). After 3 days, flow cytometry was performed (Figure 3G). PMN‐ and M‐MDSCs were isolated from the lungs of mice that had received bleomycin or normal saline 3, 7, 14, or 21 days earlier. The PMN‐ and M‐MDSCs from the bleomycin and control group were then individually co‐cultured with CD4^+^ T cells. The suppressive activity of the PMN‐ and M‐MDSCs from the bleomycin and control group was then evaluated on the different days. The suppressive activity of PMN‐MDSCs from bleomycin‐treated mice was mainly pronounced on day 3, but M‐MDSCs retained significant suppressive activity from day 3 to day 21 (Figure 3H–O). No significant differences were observed in the suppressive activity of the PMN‐MDSCs of the bleomycin‐treated group compared to those of the control group on any day after bleomycin treatment. However, the PMN‐MDSCs in the group receiving bleomycin showed a trend towards higher suppressive activity on day 3, which decreased over the course of 21 days (Figure 3H–K). The M‐MDSCs of the bleomycin group were significantly more suppressive on day 3 than those of the control group (Figure 3L). They retained their suppressive potential and showed significantly increased suppressive activity up to day 14, before approaching the control group on day 21 (Figure 3M–O).

### 3.5 T Cell Abundance Peaked 7 Days After Bleomycin Challenge

**FIGURE 4:**
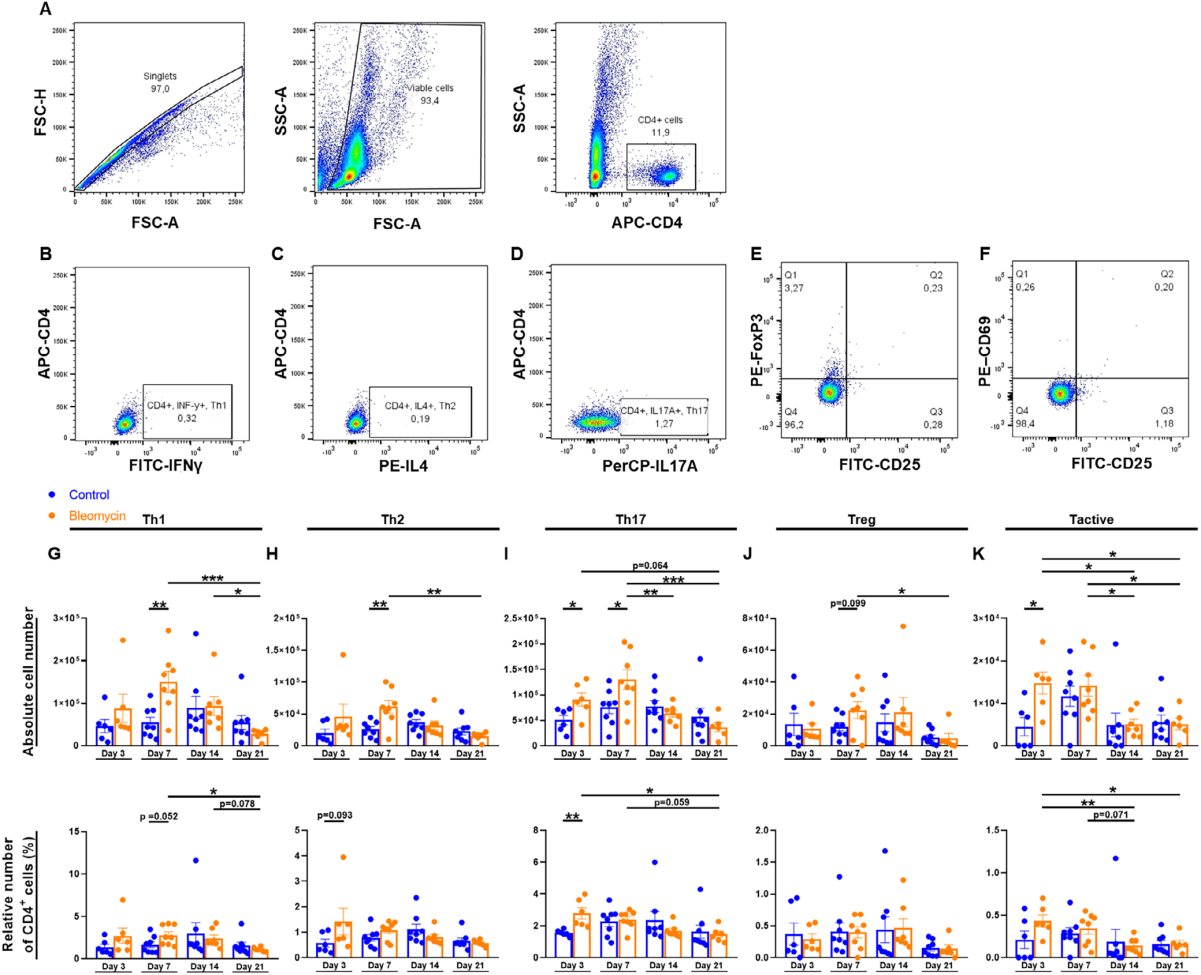
The effect of a murine model of bleomycin‐induced pulmonary fibrosis on pulmonary T cell subsets on days 3, 7, 14, and 21. 9‐ to 12‐week‐old C57BL/6N mice received either 50 μL of 0.9% normal saline or 50 μL of 1.5 U/kg bleomycin i.t. on day 0. On days 3, 7, 14, and 21, lung cells were isolated for quantitative analysis of different T cell subsets. Their number was assessed by flow cytometry. (A) After gating for CD4^+^ cells, T helper cells were characterized as (B) Th1 (CD4^+^IFNγ^+^), (C) Th2 (CD4^+^IL‐4^+^), (D) Th17 (CD4^+^IL‐17A^+^), (E) T regulatory cells (CD4^+^CD25^+^FoxP3^+^) and (F) T active cells (CD4^+^CD25^+^CD69^+^). Absolute and relative numbers of CD4^+^ cells are shown for (G) Th1 cells, (H) Th2 cells, (I) Th17 cells, (J) T regulatory cells, and (K) T active cells. Data was obtained from 8 independent experiments, *n* = 6–8. Results are shown as mean ± SEM. Statistical analysis was performed within the control and the bleomycin group between different days with one‐way ANOVA followed by Tukey's multiple comparisons test or with Kruskal–Wallis test followed by Dunn's multiple comparisons test. For comparison of the control and the bleomycin group of the same day, unpaired two‐tailed Student's t‐tests or Mann–Whitney test was used. If data followed normal distribution, one‐way ANOVA or unpaired two‐tailed Student's t‐tests were performed. Otherwise, Kruskal–Wallis test or Mann–Whitney test was used. Significance was defined as follows: *p < 0.05, **p < 0.005, ***p < 0.001. Nonsignificant results are listed if *p*‐value is > 0.05 and ≤ 0.099.

The number of T helper cells (Th) 1, Th2, Th17, regulatory T cells, and activated T cells of the lung was determined by flow cytometry on days 3, 7, 14, and 21 to assess the alterations of different T cell subsets after bleomycin administration (Figure 4A–F). Th1 and Th2 cells showed similar dynamics. Notably, the bleomycin‐treated group exhibited a significant increase in Th1 and Th2 cells by day 7, which was followed by a reduction in cell numbers towards day 21 (Figure 4G,H). Similarly, Th17 cells exhibited a comparable pattern but displayed a significant increase within the bleomycin group as early as day 3 compared to the control group (Figure 4I). Regulatory T cells showed a trend towards an initial increase until day 7, followed by a decrease in the bleomycin group (Figure 4J). In contrast, activated T cells exhibited a different pattern, with a significant increase in the absolute cell number within the bleomycin group already on day 3, followed by a decline until day 21 (Figure 4K).

### 3.6 Adoptive Transfer of MDSCs Did Not Lead to Significant Changes in Lung Function In Vivo

**FIGURE 5:**
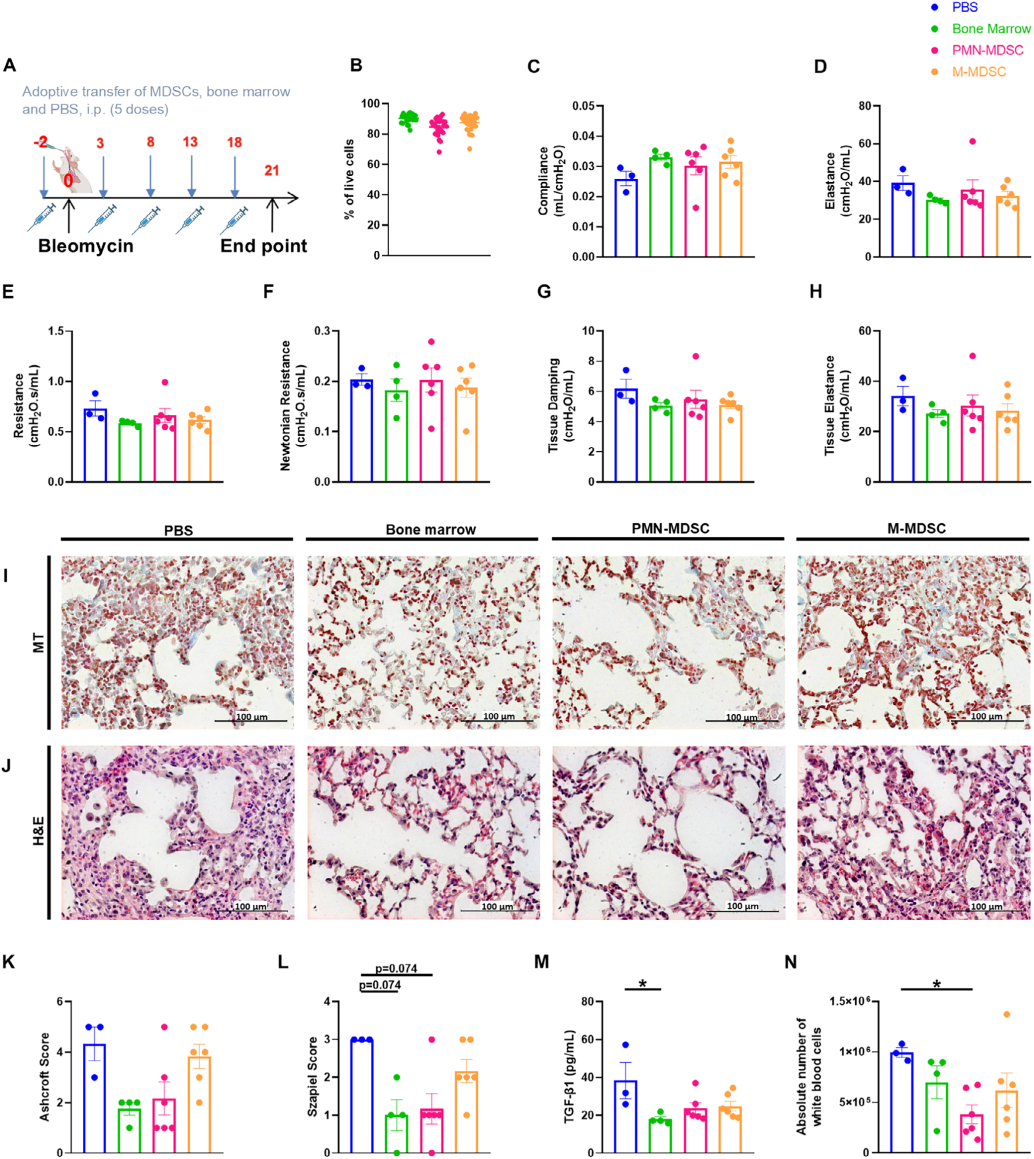
The effect of adoptive transfer of MDSCs on in vivo lung function measurements, inflammation and fibrosis development. (A) 9‐ to 12‐week‐old C57BL/6N mice received 1.5 U/kg bleomycin i.t. on day 0. Adoptive transfer was performed five times. On day 21 lung function and immunological parameters were investigated. (B) Purity of bone marrow cells, PMN‐ and M‐MDSCs of mice which have been challenged with 1.5 U/kg bleomycin 3 days before cell isolation. (C–H) Various parameters were obtained during an in vivo lung function measurement, including (C) compliance (C_rs_), (D) elastance (E_rs_), (E) resistance (R_rs_), (F) Newtonian resistance (R_n_), (G) tissue damping (G) and (H) tissue elastance (H). (I and J) Representative histological images (20× magnification) stained with (I) MT or (J) H&E. (K) Pulmonary fibrosis was assessed with the Ashcroft score. (L) Alveolar inflammation was assessed using the Szapiel score. (M and N) BALF was collected. (M) TGF‐β1 concentration was determined with an ELISA from BALF supernatant. (N) Absolute number of white blood cells. Data was obtained for (B) from 13 to 15 independent experiments, *n* = 2, for (C–N) from 3 independent experiments, *n* = 3–6. Results are shown as mean ± SEM. Statistical analysis was performed with one‐way ANOVA followed by Tukey's multiple comparisons test if the data were normally distributed, otherwise Kruskal–Wallis test followed by Dunn's multiple comparisons test was applied. Significance was defined as follows: *p < 0.05. Nonsignificant results are listed if *p*‐value is > 0.05 and ≤ 0.099.

Since the suppressive activity of PMN‐ and M‐MDSCs was most pronounced on day 3 (Figure 3H,L), we decided to perform further experiments with adoptively transferred PMN‐ and M‐MDSCs isolated from mice 3 days after bleomycin administration. In order to examine the effect of adoptive transfer on lung function of bleomycin‐treated mice in vivo, lung function measurement was performed 21 days after bleomycin administration. Over this period, adoptive transfer of PMN‐, M‐MDSCs, or bone marrow cells was carried out five times (Figure 5A). Bone marrow cells, PMN‐, and M‐MDSCs were isolated with purities of 90.4 ± 2.8%, 84.7 ± 5.7%, and 87.6 ± 5.2%, respectively (Figure 5B). We used bone marrow cells as a positive control to the isolated PMN‐ and M‐MDSCs. Overall, almost all measured parameters, with the exception of Newtonian resistance, showed a slight improvement in lung function in all adoptive transfer groups compared to the PBS group. However, none of these changes proved to be significant (Figures 5C–H and S2A–F). Nevertheless, for quasi‐static compliance and quasi‐static elastance, this improvement was significant in the group receiving adoptive transfer of bone marrow cells (Figure S2A,B). These results were emphasized by the results of the PV curves, which showed a rightward shift that was most pronounced in the PBS group, followed by the PMN‐ and the M‐MDSCs groups (Figure S2F).

### 3.7 Adoptive Transfer of PMN‐MDSCs and Bone Marrow Cells Attenuated Inflammation and Fibrosis Development

To assess the effect of adoptive transfer on histopathological changes, the diaphragmatic lobe of the left lung was collected 21 days after bleomycin challenge, and the respective adoptive transfer protocol and MT and H&E staining were carried out (Figure 5A). In the groups receiving adoptive transfer of PMN‐MDSCs and bone marrow cells, a decrease in inflammation and fibrosis development was observed compared to the PBS group, as reflected by lower Szapiel and Ashcroft score values (Figure 5I–L). Only a slight reduction in values was evident in the group receiving M‐MDSCs compared to the PBS group (Figure 5K,L). Notably, the attenuated fibrosis development and inflammation in the PMN‐MDSC and the bone marrow groups were visible in MT staining (Figure 5I) and H&E staining (Figure 5J). A similar trend emerged in an ELISA performed with BALF collected 21 days after bleomycin administration. Compared to the PBS group, adoptive transfer of PMN‐, M‐MDSCs, and bone marrow cells showed a trend towards a reduction in TGF‐β1 levels, with this change being significant for bone marrow cells (Figure 5M). BALF was also used to investigate the impact of adoptive transfer on white blood cells in the respiratory tract. Regarding the absolute number of white blood cells, a significant reduction was observed in the PMN‐MDSCs group compared to the PBS group (Figure 5N). All adoptive transfer groups showed a trend towards lower absolute cell numbers in the differential white blood cell counts compared to the PBS group (Figure S2G–J). In the bone marrow group, this decrease was even significant for the relative number of lymphocytes (Figure S2I).

### 3.8 MDSC and T Cell Numbers Were Increased Following Adoptive Transfer of PMN‐MDSCs

To examine the impact of adoptive transfer on the generation of MDSCs, the number of PMN‐MDSCs and M‐MDSCs in the lung and in the spleen was assessed by flow cytometry on day 21 after bleomycin challenge and the respective adoptive transfer protocol (Figure 5A). Regarding the absolute cell number in the lung and spleen, for both PMN‐ and M‐MDSCs, there was a tendency towards an increased cell number in the group that received adoptive transfer of PMN‐MDSCs compared to the PBS group (Figure S3A–D). A similar pattern emerged for the number of T cells in the lung, which were determined by flow cytometry. The group subjected to PMN‐MDSC adoptive transfer showed a trend towards an increased absolute cell number of Th1, Th2, Th17, and regulatory T cells compared to the PBS group (Figure S3E–H). The group receiving adoptive transfer of M‐MDSCs displayed a trend towards an increased number of Th1, Th17, and T active cells compared to the PBS group (Figure S3E,G,I).

### 3.9 PMN‐MDSC Depletion Did Not Lead to an Exacerbation of Pulmonary Fibrosis

**FIGURE 6:**
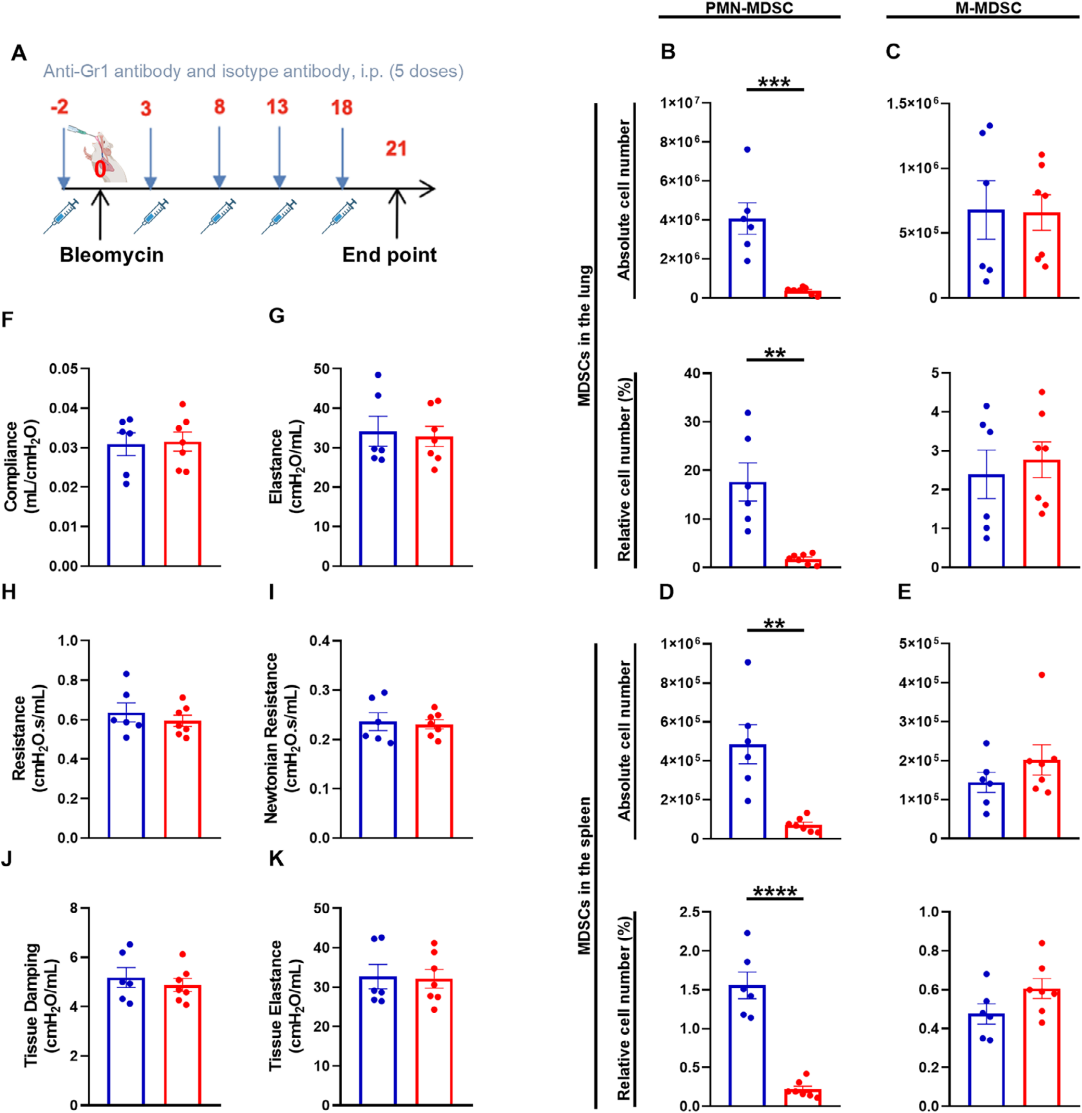
The effect of PMN‐MDSC depletion on innate MDSCs in lung and spleen and on in vivo lung function. (A) Timeline of the PMN‐MDSC depletion. 9‐ to 12‐week‐old C57BL/6N mice received 1.5 U/kg bleomycin i.t. on day 0 and five times (2 days before bleomycin application and on days 3, 8, 13 and 18) an i.p. injection of either 200 μL isotype antibody or 200 μL anti‐Gr1 antibody. On day 21 lung function and immunological parameters were investigated. Lung and spleen cells were isolated for quantitative analysis of both PMN‐ and M‐MDSC. The number of PMN‐ and M‐MDSCs was assessed by flow cytometry. Data shown as absolute cell number and as relative cell number in the lung for (B) PMN‐MDSCs, (C) M‐MDSCs and in the spleen for (D) PMN‐MDSCs and (E) M‐MDSCs. Various parameters were obtained during an in vivo respiratory lung function measurement, such as (F) compliance (C_rs_), (G) elastance (E_rs_), (H) resistance (R_rs_), (I) Newtonian resistance (R_n_), (J) tissue damping (G) and (K) tissue elastance (H). Data was obtained from 2 to 3 independent experiments with lung and spleen cells from each mouse, including two replicates per mouse, *n* = 6–7. Results are shown as mean ± SEM. Statistical analysis was performed with unpaired two‐tailed Student's t‐tests if the data were normally distributed; otherwise, Mann–Whitney test was used. Significance was defined as follows: **p < 0.005, ***p < 0.001, ****p < 0.0001.

**FIGURE 7:**
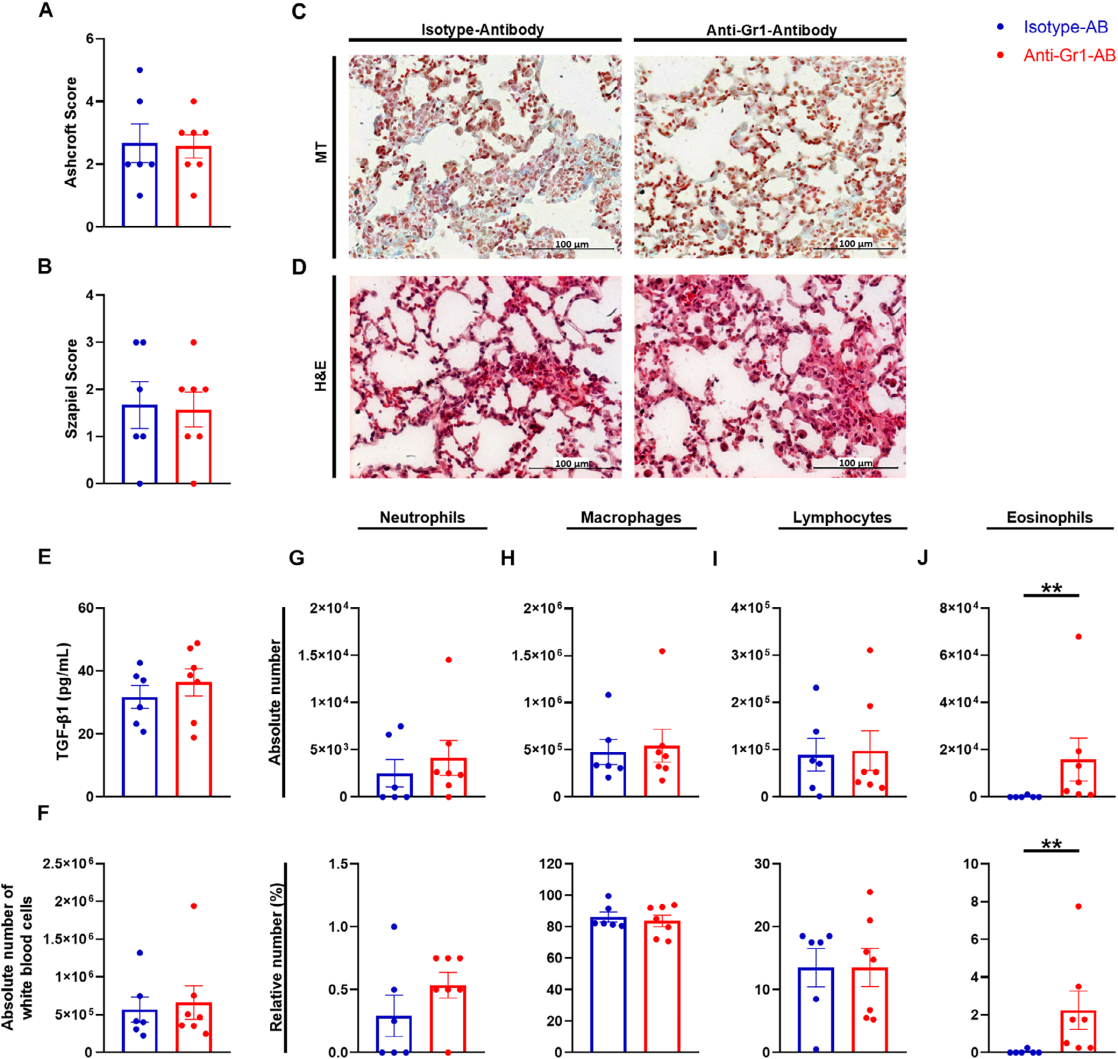
The effect of PMN‐MDSC depletion on inflammation and fibrosis. 9‐ to 12‐week‐old C57BL/6N mice received 1.5 U/kg bleomycin i.t. on day 0 and five times an i.p. injection of either 200 μL isotype antibody or 200 μL anti‐Gr1 antibody. (A–D) On day 21, the left diaphragmatic lobe was collected for histopathological analysis. (A) Pulmonary fibrosis was assessed with the Ashcroft score. (B) Alveolar inflammation was assessed using the Szapiel score. Representative histological images (20× magnification) of PMN‐MDSC depleted mice stained with (C) MT and (D) H&E. (E–J) BALF was collected and processed for differential cell count, supernatant was stored. (E) TGF‐β1 concentration was determined with an ELISA from the BALF supernatant. (F) Absolute number of white blood cells. The absolute and the relative cell number is presented for (G) neutrophils, (H) macrophages, (I) lymphocytes and (J) eosinophils. Data was obtained from 2 to 3 independent experiments, *n* = 6–7. Results are shown as mean ± SEM. Statistical analysis was performed with unpaired two‐tailed Student's t‐tests if the data were normally distributed, otherwise Mann–Whitney test was used. Significance was defined as follows: **p < 0.005.

As adoptive transfer of PMN‐MDSCs had shown the most promising results in terms of reducing inflammation and fibrosis development in the lung, we decided to deplete PMN‐MDSCs in our study to further investigate the impact of PMN‐MDSCs on fibrosis development. To analyze the effect of an anti‐Gr1 antibody on MDSCs in vivo, the number of PMN‐MDSCs and M‐MDSCs in the lung and in the spleen was assessed by flow cytometry on day 21 after bleomycin administration. During this period, five i.p. applications of an anti‐Gr1 antibody were administered in order to deplete PMN‐MDSCs (Figure 6A). In both lung and spleen, the depletion of PMN‐MDSCs was evident within the group receiving an anti‐Gr1 antibody (Figure 6B,D). In M‐MDSCs, no significant change was observed in the lung and spleen between the anti‐Gr1 antibody‐treated group and the isotype antibody‐treated group (Figure 6C,E). Moreover, in vivo lung function measurement performed 21 days after bleomycin administration revealed no differences between the two groups, suggesting that PMN‐MDSC depletion had no discernible effect on in vivo respiratory parameters (Figures 6F–K and S4A–F). In addition, depletion of PMN‐MDSCs did not alter Szapiel or Ashcroft scores, indicating no changes in inflammation or fibrosis development compared to the group receiving an isotype antibody (Figure 7A–D). A slight trend towards an increase in the TGF‐β1 concentration was observed in the group receiving an anti‐Gr1 antibody. However, this did not prove to be significant (Figure 7E). In the BALF, a significant increase of eosinophils was evident in the PMN‐MDSC depleted group (Figure 7J). Regarding the absolute number of neutrophils, a trend towards an increase was observed in the anti‐Gr1 antibody group (Figure 7G). However, there were no observable differences regarding the absolute number of white blood cells and in macrophages and lymphocytes number between the isotype and anti‐Gr1 antibody‐treated groups (Figure 7F,H,I). Thus, depletion with an anti‐Gr1 antibody did not lead to changes in histopathological features of pulmonary fibrosis. Furthermore, the number of T cells from the lung was assessed by flow cytometry. In the group treated with an anti‐Gr1 antibody, a slight tendency towards an elevation in Th2 and T active cells was discernible (Figure S4H,K). For Th1, Th17 and regulatory T cells, the cell count was similar to the non‐PMN‐MDSC depleted group (Figure S4G,I,J). Therefore, PMN‐MDSC depletion by an anti‐Gr1 antibody had no significant effect on various T cell subsets.

## 4 Discussion

In this study, we examined the role of MDSCs in a murine model of bleomycin‐induced pulmonary fibrosis. In particular, we investigated the effects of their immunosuppressive activity on the transition from inflammation to fibrosis.

The detrimental effect of bleomycin on lung function has been well documented [24, 25, 26, 27, 28]. We were able to demonstrate respiratory mechanics typical of pulmonary fibrosis that worsened as the disease progressed. In addition, we could show that different cell types predominate in the BALF during the different phases of pulmonary fibrosis. Furthermore, we observed increased TGF‐β1 levels among bleomycin‐treated mice. These changes were consistent with the increased presence of inflammation and fibrosis in the lungs.

It is known that pathological conditions such as cancer, inflammation, and infection lead to an expansion of MDSCs with immunosuppressive functions [13]. However, there are still many uncertainties about the occurrence of MDSCs in pulmonary fibrosis. Our aim was to investigate the alterations in MDSCs during the acute inflammatory phase and the transition to the chronic phase. The activation, generation, and activity of MDSCs appeared to be different in the acute lung injury and chronic stages. Days 3 and 7 were defined as the acute inflammatory stage, while days 14 and 21 corresponded to the chronic fibrosis stage. Inflammation was observed from day 3, while fibrosis was seen from day 7. However, some of the first fibrosis‐typical changes were already detected on day 3. On days 3 and 7, the number of PMN‐MDSCs in the lung was similar or lower in the bleomycin group than in the control group. Thereafter, in the chronic phase, there appeared to be an increase in cell numbers, while suppressive activity appeared to decrease. In contrast, M‐MDSCs in the lung showed a significantly increased number of cells and a strong suppressive activity in the acute inflammatory stage. It is known that when assessed on a per‐cell basis, M‐MDSCs are more suppressive than PMN‐MDSCs [29]. In addition, the in vivo lifespan of MDSCs is only a few days [30]. Precisely, PMN‐MDSCs are known to have a shorter lifespan than M‐MDSCs [29, 31]. Since we assessed the cell number in the lung for the first time on day 3, we only obtained a section of the dynamics of the MDSCs. It is possible that PMN‐ or M‐MDSCs were already recruited to the lung before day 3. We observed an increased number of M‐MDSCs at that point, which could mean that they were either recruited to the lung in the first 3 days and remained there longer due to their longer survival time, or that they were only recruited into the lung on day 3. In contrast, we observed no increased number of PMN‐MDSCs on day 3. However, there is a possibility that there may have already been a short‐lived spike in the recruitment of PMN‐MDSCs preceding day 3, which was followed by a rapid decline due to the aforementioned shorter lifespan. Nevertheless, we can only assume that both subgroups were in the lungs during the acute phase of the inflammation. The recruitment of a new generation of PMN‐MDSCs to the lung could explain the small increase in the number of PMN‐MDSCs observed in the chronic phase. The chronic phase is in line with increased inflammatory activity and the augmented presence of fibrosis. The amplified development of fibrosis from day 7 could therefore appear to promote the recruitment of PMN‐MDSCs into the lung. In addition, especially in the chronic phase, there was a recruitment of PMN‐ and M‐MDSCs to the spleen. However, an increase was also observed in the control group. The reason for the increase in the control group and the role of PMN‐MDSCs in the lung in the chronic phase needs to be further investigated.

T cells play a crucial role in the development of fibrosis, as they can have both pro‐ and antifibrotic effects [32, 33, 34, 35, 36]. We were able to show that the distribution of the different T cell subsets was similar in the early and later stages of pulmonary fibrosis, with the highest number of all T cell subsets always being reached in the early inflammatory phase. This underlines their important role in the development of fibrosis.

To further investigate the effect of MDSCs on pulmonary fibrosis, an adoptive transfer was performed. We hypothesized that the anti‐inflammatory properties of adoptively transferred MDSCs would dampen the inflammatory and fibrosis response. Previous studies have shown that adoptive transfer of MDSCs led to an improvement in various inflammatory diseases, e.g., asthma [37, 38, 39]. In addition, Hsieh et al. demonstrated that adoptive transfer of cytokine‐induced MDSCs attenuated renal fibrosis in diabetic mice [40]. We were able to show histologically that inflammation and fibrosis development slightly decreased after adoptive transfer of PMN‐MDSCs and bone marrow cells. Previous data have shown that mobilizing cells from the bone marrow could ameliorate bleomycin‐induced pulmonary fibrosis [41, 42, 43]. However, further studies are required to identify the cell subset from the adoptively transferred bone marrow that leads to improvement of fibrosis. Su et al. reported that the transfer of bone marrow mesenchymal stem cells (BMSC) into bleomycin‐treated mice resulted in the differentiation of inflammatory Gr1^High^CD11b^+^ cells, which expressed neutrophilic markers and lacked immunosuppressive activity, into immunosuppressive Gr1^Low^CD11b^+^ cells, presumably M‐MDSCs [44]. They indicated that BMSC could be involved in MDSC differentiation by M‐CSF and the resulting expansion of monocytic Gr1^Low^CD11b^+^ cells [44]. They also suggested that the paracrine release of growth factors and anti‐inflammatory cytokines by BMSC, or the release triggered by them, could be an important factor in tissue repair. This could even play a greater role than the replacement of damaged lung cells by donor cells [44]. However, we cannot say with certainty whether a similar differentiation took place in our study. Further experiments are needed to determine the effects and mechanisms that bone marrow has on MDSCs. It is known that the activity of MDSCs is related to the environment [13, 37]. We do not know to what extent the complex in vivo conditions affected the immunosuppressive activity of adoptively transferred MDSCs and whether the expression of the marker profile of adoptively transferred MDSCs could have been altered after adoptive transfer [37, 45]. The timing and frequency of the adoptive transfer as well as the number of MDSCs might also play an important role [37]. Taken together, our results indicate that adoptive transfer of PMN‐MDSCs and bone marrow cells slightly attenuated inflammation and fibrosis development in the lung. The adoptive transfer of M‐MDSCs seems to have lower attenuating effects compared to the adoptive transfer of PMN‐MDSCs or bone marrow cells. This is an interesting observation considering that M‐MDSC showed stronger suppressive activity than PMN‐MDSCs in previous experiments (Figure 3H–O).

Since adoptive transfer of PMN‐MDSCs had shown more promising results than adoptive transfer of M‐MDSCs, we next depleted PMN‐MDSCs with an anti‐Gr1 antibody to determine whether this would exacerbate inflammation and fibrosis development. Previous data indicated that depletion of MDSCs often had the opposite effect to adoptive transfer [46, 47, 48]. Anti‐Gr1 antibodies are widely used to deplete Gr1^+^ cells such as MDSCs [46, 49]. The anti‐Gr1 antibody used for the PMN‐MDSC depletion experiments specifically binds to Ly6G, a surface marker of PMN‐MDSCs and neutrophils [46, 49]. MDSCs are mainly defined by various cell surface markers and their ability to suppress T cells [50]. In mice, the markers CD11b^+^Ly6C^lo^Ly6G^+^ are used to identify PMN‐MDSC and CD11b^+^Ly6C^hi^Ly6G^−^ to identify M‐MDSC [14]. However, due to their phenotypic similarity to neutrophils and monocytes, it is not sufficient to characterize MDSCs based on the markers alone [50]. Differentiation of MDSCs from neutrophils or monocytes has so far mainly been based on markers, functional activity, and biochemical characteristics [51]. Measuring T cell suppression for evaluating the functional properties of MDSCs in combination with the expression of the phenotypic markers described above appears to be sufficient to define cells as MDSCs [50, 51]. Measuring suppression in vivo and determining whether MDSCs have other functions in addition to their immunosuppressive activity could be helpful in the future [51]. Recent evidence from studies investigating neutrophils in cancer suggests that neutrophils have two main functional states: classically activated neutrophils and pathologically activated immunosuppressive MDSCs [52]. Even though these have different characteristics, it remains a challenge to distinguish and specifically target them [52]. Although the use of an anti‐Gr1 antibody has its limitations, it can be a useful tool to further elucidate the role of MDSCs. In combination with other approaches such as adoptive transfer, it provides useful information on the contribution of MDSCs to various disease conditions [46, 49]. Since the antibodies only have a short duration of action of a few days and a rebound of Gr1^+^ cells can occur, repeated application is necessary [49, 53]. After repeated treatment with an anti‐Gr1 antibody, we observed low cell counts of PMN‐MDSCs, but not M‐MDSCs in the lung and spleen, suggesting that depletion of Gr1^+^Ly6G^+^ PMN‐MDSCs was successful. Nevertheless, depletion of PMN‐MDSCs did not lead to a worsening of fibrosis as might have been observed with a deterioration in in vivo lung function or with increased inflammation. We detected no differences in in vivo lung function measurements or in the development of inflammation or fibrosis. However, we observed some reversible effects after PMN‐MDSC depletion compared to PMN‐MDSC adoptive transfer. While depletion of PMN‐MDSC showed a trend towards increased TGF‐β1 levels, adoptive transfer led to a trend towards reduced TGF‐β1 levels. In addition, adoptive transfer of PMN‐MDSCs and bone marrow cells showed a slight decrease in inflammation and fibrosis, which was reflected in the absolute number of white blood cells and in histology. Interestingly, both adoptive transfer of PMN‐MDSCs and depletion of PMN‐MDSCs tended to increase different T cell subsets. However, it should be emphasized that the role of T cells in pulmonary fibrosis is very complex and not yet fully understood. In addition, the interaction of the various T cell subgroups with each other has also not yet been sufficiently clarified. It is also possible that the repeated i.p. injections for adoptive transfer and PMN‐MDSC depletion have led to a possible increase. Furthermore, Haverkamp et al. conducted a study in which deletion of PMN‐MDSCs by deletion of myeloid leukemia cell‐1 led to an increase in M‐MDSCs that effectively compensated for the loss of PMN‐MDSCs [14, 54]. In contrast to their results, we did not observe an increase of M‐MDSCs. Overall, we were able to show that an anti‐Gr1 antibody successfully depleted PMN‐MDSCs. However, this did not lead to an exacerbation of pulmonary fibrosis. Although our results suggest that MDSCs play an important role in the pathogenesis of pulmonary fibrosis, it must be emphasized that we often observed only minor changes. We do not know whether this is solely due to the effect of MDSCs or whether other (confounding) factors may also play a role. Furthermore, the inflammatory response and the development of fibrosis are complex and mediated by multiple mechanisms, many of which are not yet fully understood [49].

The objective of our study was to investigate the role of MDSCs in a murine model of bleomycin‐induced pulmonary fibrosis. We demonstrated that inflammation and fibrosis increased from day 3 after bleomycin administration. This increase correlated with a deterioration of lung function in vivo, especially from day 7 onwards. The changes in the early inflammatory phase were accompanied by a low number of PMN‐MDSCs and a high number of M‐MDSCs in the lung. Especially, M‐MDSCs showed enhanced suppressive activity on day 3. Furthermore, the adoptive transfer of bone marrow and PMN‐MDSCs attenuated inflammation and fibrosis development. However, PMN‐MDSC depletion did not lead to an exacerbation of pulmonary fibrosis. In summary, our findings suggest that PMN‐ and M‐MDSCs elicit different patterns in terms of number and activity depending on the acute inflammatory and fibrosis stage in a bleomycin‐induced pulmonary fibrosis model in mice. We could not fully prove that MDSCs are able to attenuate inflammation and thus the transition to fibrosis. However, we were able to show that the adoptive transfer of bone marrow cells and PMN‐MDSCs could partially ameliorate inflammation and fibrosis. Nevertheless, further studies are necessary to fully elucidate the mechanisms in detail.

## Author Contributions

Conceptualization: Saeed Kolahian. Methodology: Saeed Kolahian. Investigation: Nora Vedder, Philipp Gercke, Nikoleta Lautenschlager, Tobias Brunn, Tim Lange, Jakob Schieb, Charlotte Vetter, Chiel van Geffen, and Saeed Kolahian. Visualization: Nora Vedder. Supervision: Philipp Gercke, Chiel van Geffen, and Saeed Kolahian. Writing – original draft: Nora Vedder. Writing – review and editing: Nora Vedder, Philipp Gercke, Chiel van Geffen, and Saeed Kolahian.

## Ethics Statement

Experiments using animals were approved by the Regierungspräsidium Tübingen, Germany, and by the Regierungspräsidium Giessen, Germany (protocol G8/2022) and were carried out according to Directive 2010/63/EU and German legislation.

## Conflicts of Interest

The authors declare no conflicts of interest.

## Supporting information


Figure S1.

## Data Availability

All data needed to evaluate the conclusions in the paper are present in the paper and the Supporting Information. The data can be provided by Dr. Saeed Kolahian pending scientific review and a completed material transfer agreement. Requests for the data should be submitted to: saeed.kolahian@uni-marburg.de.
