# Supplementary material for: Characterizing the Emergence of Myeloid‐Derived Suppressor Cell Subsets in a Murine Model of Pulmonary Fibrosis
Source: FASEB J. 2025 May 13;39(10):e70626. doi: 10.1096/fj.202500312RR (PMC12070151; doi:10.1096/fj.202500312RR)
Supplement: Supplementary file 1 — Figure S1. [file FSB2-39-e70626-s001.pdf]

Supplementary Material for:

“Characterizing the emergence of myeloid-derived suppressor cell subsets in a murine model of pulmonary fibrosis”

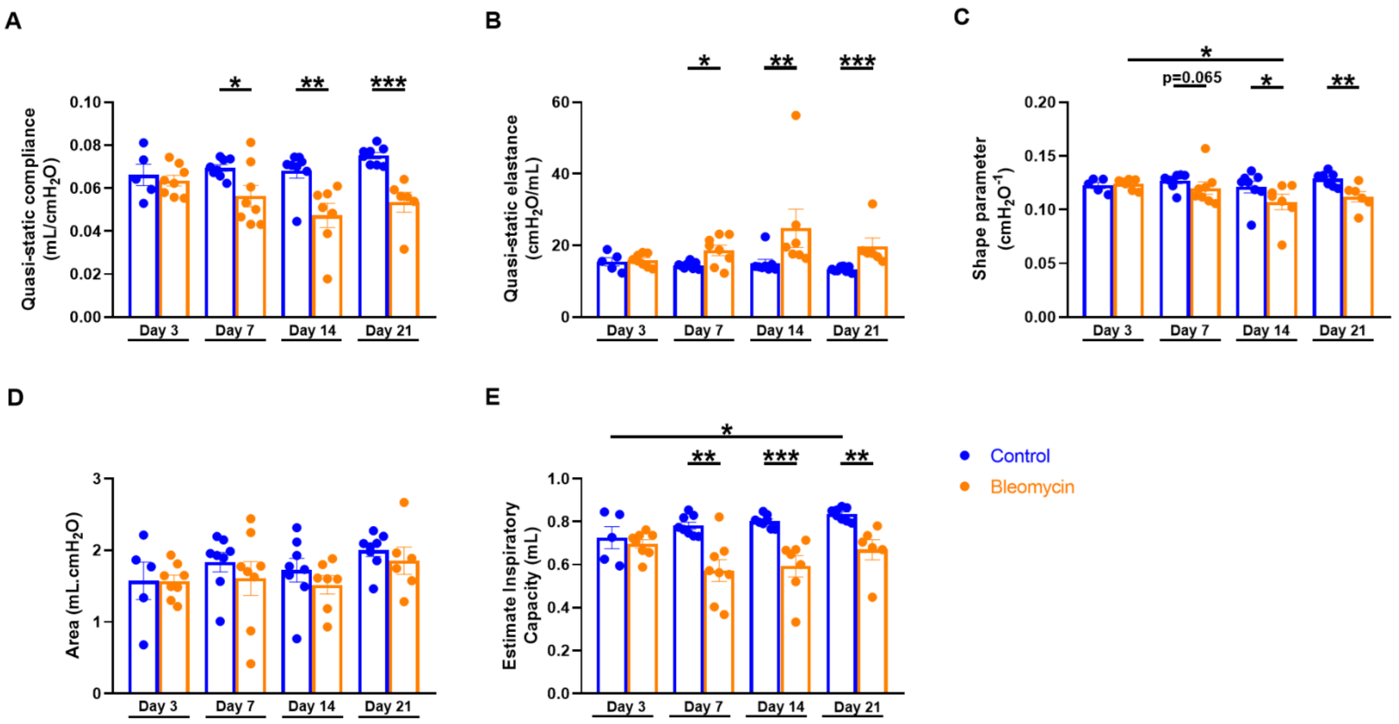

Fig. S1. The effect of a murine model of bleomycin-induced pulmonary fibrosis on other parameters of an *in vivo* lung function measurement. Nine- to twelve-week-old C57BL/6N mice received either 50 µl of 0.9 % normal saline or 50 µl of 1.5 U/kg bleomycin i.t. on day 0. An *in vivo* respiratory lung function measurement was performed on days 3, 7, 14 and 21. Various parameters were obtained such as **(A)** Quasi-static compliance (C_{st}), **(B)** Quasi-static elastance (E_{st}), **(C)** Shape parameter (K), **(D)** Area and **(E)** Estimate of the Inspiratory Capacity (A). Data was obtained from 8 independent experiments, n=5-8. Results are shown as mean ± SEM. Statistical analysis was performed with one-way ANOVA followed by Tukey’s multiple comparisons test or unpaired two-tailed Student’s t-tests if the data were normally distributed. Otherwise Kruskal-Wallis test followed by Dunn’s multiple comparisons test or Mann-Whitney test was applied. Significance was defined as follows: *$p<0.05$, **$p<0.005$, ***$p<0.001$. Nonsignificant results are listed if p-value is >0.05 and ≤0.099.

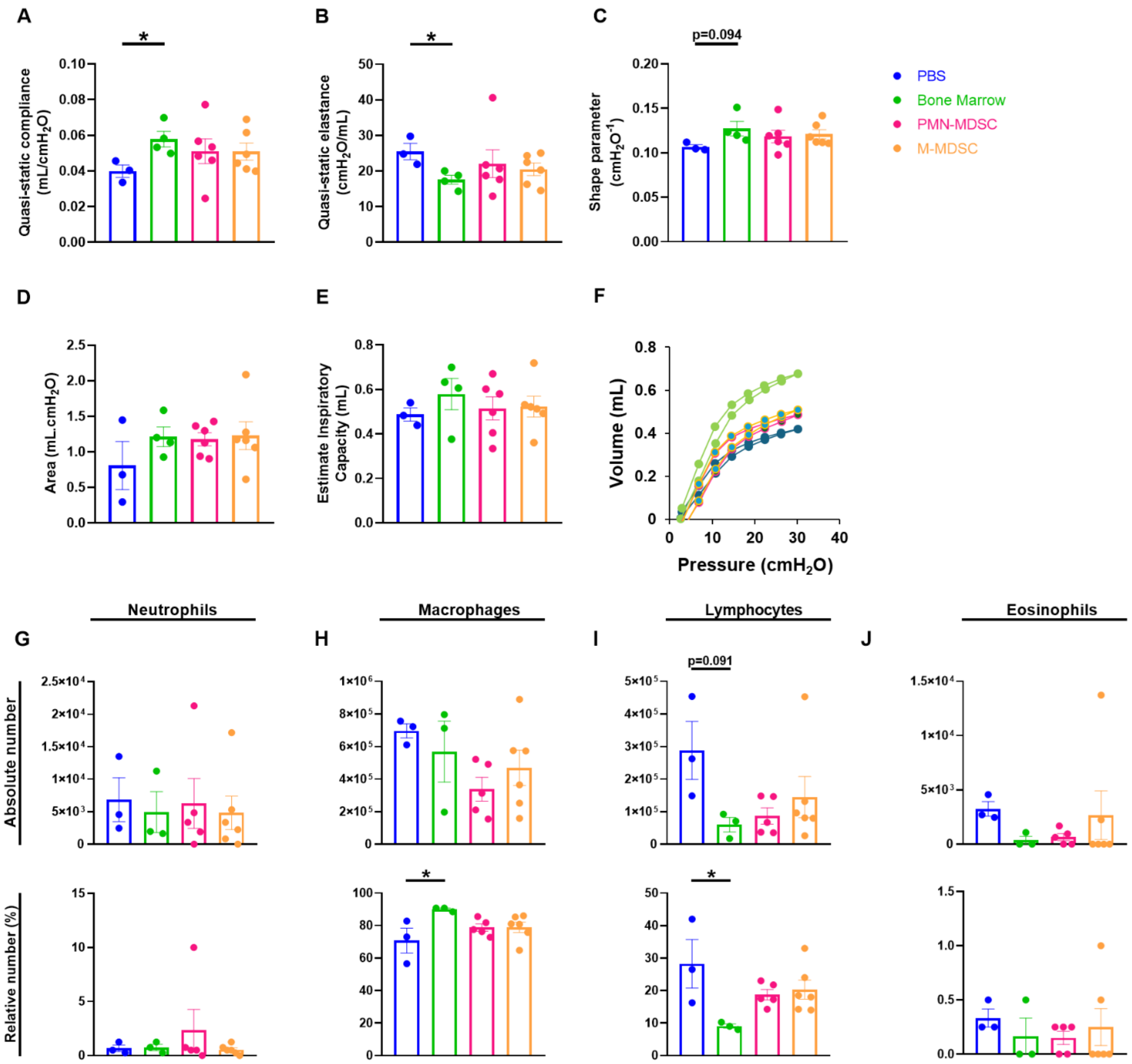

Fig. S2. The effect of adoptive transfer of MDSCs on other parameters of an *in vivo* lung function measurement and white blood cells. Nine- to twelve-week-old C57BL/6N mice received 1.5 U/kg bleomycin i.t. on day 0. Adoptive transfer with either PBS, bone marrow cells, PMN- or M-MDSCs was performed five times (2 days before bleomycin administration and on days 3, 8, 13 and 18). The results of both the $2x10^5$ and $2x10^6$ bone marrow adoptive transfer are combined in one diagram. **(A-F)** An *in vivo* respiratory lung function measurement was performed on day 21. Various parameters were obtained such as **(A)** Quasi-static compliance (C_{st}), **(B)** Quasi-static elastance (E_{st}), **(C)** Shape parameter (K), **(D)** Area, **(E)** Estimate of the Inspiratory Capacity (A) and **(F)** representative PV curve. **(G-J)** BALF was collected and processed for differential cell count on day 21. Absolute and relative cell number is presented for **(G)** Neutrophils, **(H)** Macrophages, **(I)** Lymphocytes and **(J)** Eosinophils. Data was obtained from 3 independent experiments, n=3-6. Results are shown as mean ± SEM. Statistical analysis was performed with one-way ANOVA followed by Tukey's multiple comparisons test if the data were normally distributed, otherwise Kruskal-Wallis test followed by Dunn's multiple comparisons test was applied. Significance was defined as follows: *p<0.05. Nonsignificant results are listed if *p*-value is >0.05 and ≤0.099.

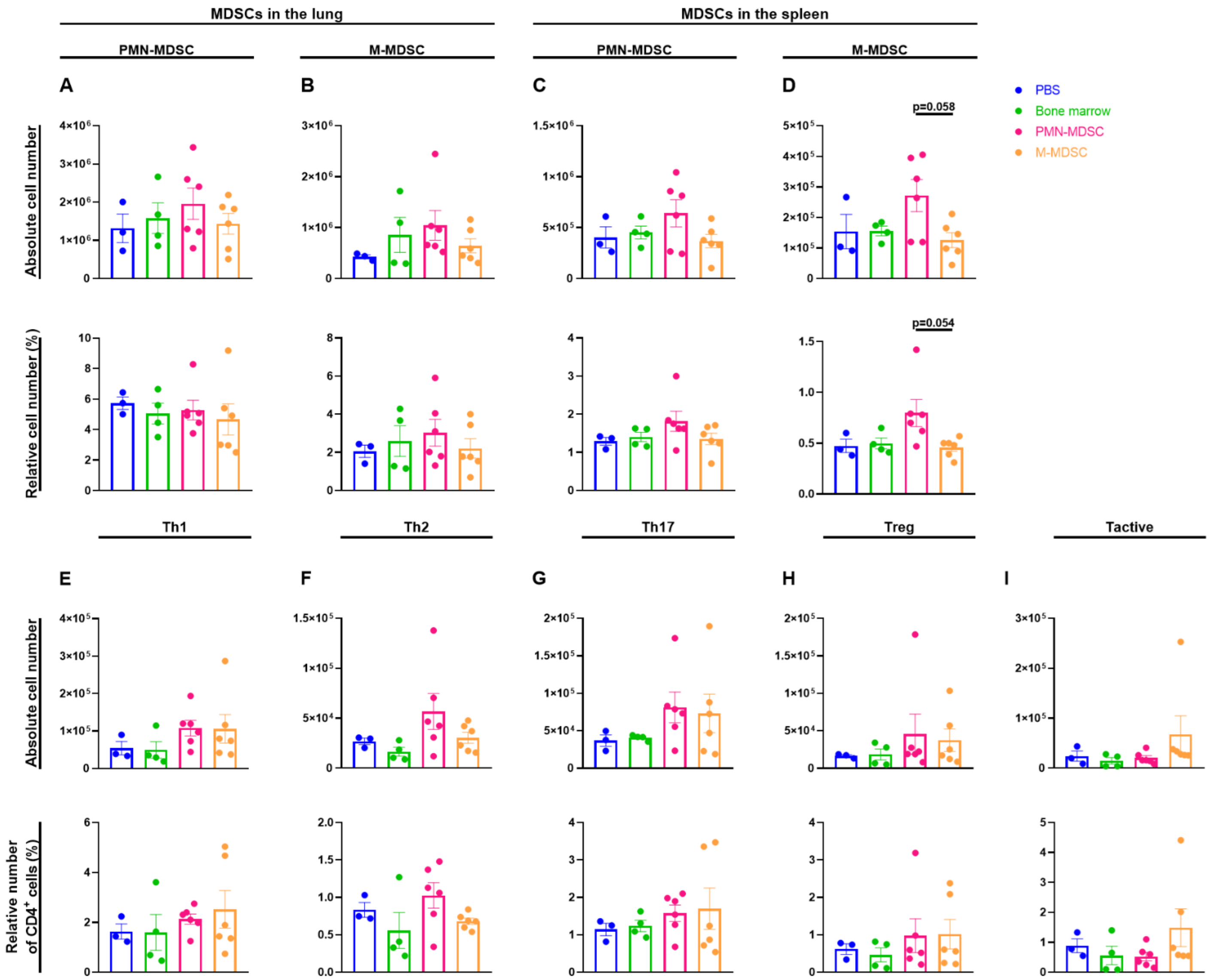

Fig. S3. The effect of adoptive transfer on innate MDSCs in lung and spleen and pulmonary T cell subsets. Lung and spleen cells were isolated from nine- to twelve-week-old C57BL/6N mice. Mice received 1.5 U/kg bleomycin i.t. on day 0. Adoptive transfer was carried out five times (2 days before bleomycin administration and on days 3, 8, 13 and 18). Mice were injected i.p. with either PBS, bone marrow cells, PMN- or M-MDSCs. The results of both the 2x10^5 and 2x10^6 bone marrow adoptive transfer are combined in one diagram. Quantitative analysis of both PMN- and M-MDSC was performed on day 21. The number of PMN- and M-MDSCs was assessed by flow cytometry. Data shown as absolute cell number and as relative cell number in the lung for **(A)** PMN-MDSCs, **(B)** M-MDSCs and in the spleen for **(C)** PMN-MDSCs and **(D)** M-MDSCs. Absolute cell number and relative cell number of CD4$^+$ lung cells are shown for **(E)** Th1 cells (CD4$^+$IFNγ$^+$), **(F)** Th2 cells (CD4$^+$IL-4$^+$), **(G)** Th17 (CD4$^+$IL-17A$^+$), **(H)** T regulatory cells (CD4$^+$CD25$^+$FoxP3$^+$) and **(I)** T active cells (CD4$^+$CD25$^+$CD69$^+$). Data was obtained from 3 independent experiments with lung and spleen cells from each mouse, including two replicates per mouse, n=3-6. Results are shown as mean ± SEM. Statistical analysis was performed with one-way ANOVA followed by Tukey's multiple comparisons test if the data were normally distributed, otherwise Kruskal-Wallis test followed by Dunn's multiple comparisons test was applied. Nonsignificant results are listed if *p*-value is >0.05 and ≤0.099.

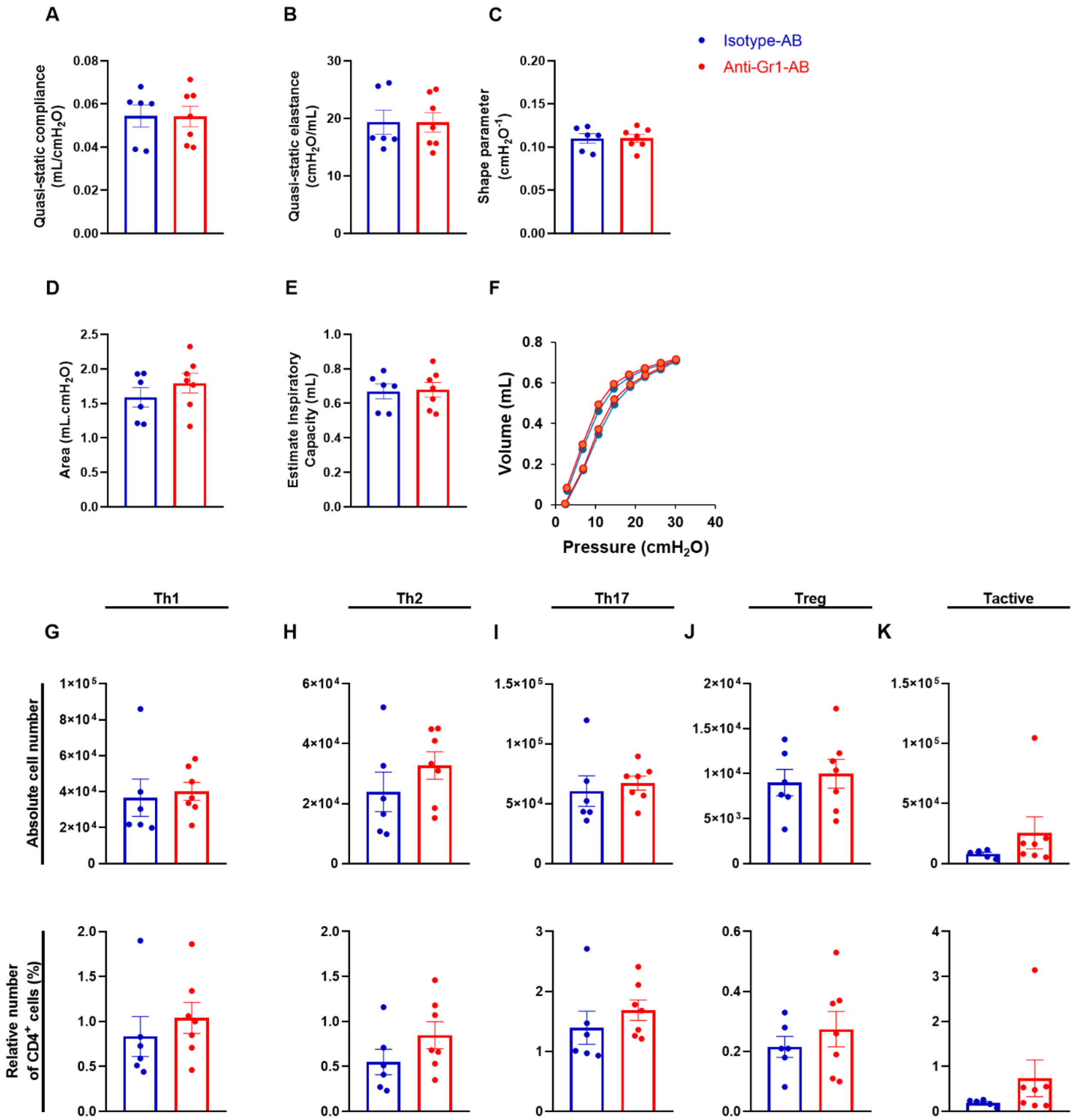

Fig. S4. The effect of PMN-MDSC depletion on other parameters of an *in vivo* lung function measurement and pulmonary T cell subsets. Nine- to twelve-week-old C57BL/6N mice received 1.5 U/kg bleomycin i.t. on day 0 and 5 times (2 days before bleomycin application and on days 3, 8, 13 and 18) an i.p. injection of either 200 µl isotype antibody or 200 µl anti-Gr1 antibody. (**A-F)** An *in vivo* respiratory lung function measurement was performed on day 21. Various parameters were obtained such as **(A)** Quasi-static compliance (C_{st}), **(B)** Quasi-static elastance (E_{st}), **(C)** Shape parameter (K), **(D)** Area, **(E)** Estimate of the Inspiratory Capacity (A) and **(F)** representative PV curve. **(G-K)** Lung cells were isolated for quantitative analysis of different T cell subsets. Their number was assessed by flow cytometry. Absolute and relative number of $CD4^+$ cells are shown for **(G)** Th1 cells ($CD4^+IFN\gamma^+$), **(H)** Th2 cells ($CD4^+IL\text{-}4^+$), **(I)** Th17 ($CD4^+IL\text{-}17A^+$), **(J)** T regulatory cells ($CD4^+CD25^+FoxP3^+$) and **(K)** T active cells ($CD4^+CD25^+CD69^+$). Data was obtained from 2-3 independent experiments, n=6-7. Results are shown as mean ± SEM. Statistical analysis was performed with unpaired two-tailed Student's t-tests if the data were normally distributed, otherwise Mann-Whitney test was used.
